# The role of pleiotrophin and β-catenin in fetal lung development

**DOI:** 10.1186/1465-9921-11-80

**Published:** 2010-06-18

**Authors:** Tingting Weng, Lin Liu

**Affiliations:** 1 Lundberg-Kienlen Lung Biology and Toxicology Laboratory, Department of Physiological Sciences, Oklahoma State University, Stillwater, Oklahoma 74078, USA

## Abstract

Mammalian lung development is a complex biological process, which is temporally and spatially regulated by growth factors, hormones, and extracellular matrix proteins. Abnormal changes of these molecules often lead to impaired lung development, and thus pulmonary diseases. Epithelial-mesenchymal interactions are crucial for fetal lung development. This paper reviews two interconnected pathways, pleiotrophin and Wnt/β-catenin, which are involved in fibroblast and epithelial cell communication during fetal lung development.

## 1. Fetal lung development

### 1.1 Stages of fetal lung development

Fetal lung development is a complex biological process which involves temporal and spatial regulation of multiple factors such as growth factors, transcriptional factors, and extracellular matrix (ECM). The development of the intimate relationship between airways and blood vessels is crucial for the normal lung function. Morphologically, mouse lung development can be divided into 5 stages: (i) Embryonic Stage (E9 to E11.5), in which lung buds originate as an outgrowth from the ventral wall of the foregut where lobar division occurs; (ii) Pseudoglandular Stage (E11.5 to E16.5), in which conducting epithelial tubes surrounded by thick mesenchyme are formed, distinguished by extensive airway branching; (iii) Canalicular stage (E16.5 to E17.5), in which bronchioles are produced, characterized by an increasing number of capillaries in close contact with cuboidal epithelium and the beginning of alveolar epithelium development; (iv) Saccular Stage (E17.5 to PN5), in which alveolar ducts and air sacs are developed; and (v) Alveolar Stage (PN5 to PN28), in which secondary septation occurs, defined by a marked increase of the number and size of capillaries and alveoli [1].

Recently, a new model of lung branching programming has been proposed, in which three branching modes govern the program of lung branching [2]. Domain branching generates daughter branches in rows along a parent branch. Planar bifurcation forms tertiary and later-generation branches with the division of a branch tip into two. Orthogonal bifurcation is composed of two cycles of plannar bifurcations with a 90° rotation between the two. These branching modes are regulated by genetically encoded subroutines, which are controlled by a master branch generator.

### 1.2 Alveolar epithelial cell differentiation

Alveolar epithelium is composed of two types of cells: alveolar epithelial type I cells (AEC I) and alveolar epithelial type II cells. In the pseudoglandular stage, columnar epithelial cells differentiate into ciliated cells with the expression of β-tubulin IV, [3] and shorter columnar cells containing large intracellular glycogen pools [4]. The latter remain undifferentiated until the canalicular stage, when some of these cells become more cuboidal AEC II and begin to synthesize and secrete surfactant. AEC II have less glycogen pools and are characterized by the appearance of lamellar bodies [5]. Some AEC II can be differentiated into AEC I.

Many transcription factors, including thyroid transcription factor-1 (TTF-1), hepatocyte nuclear factor (HNF)-3β and HNF-3/forkhead homologue-4 (HFH-4) have indispensable roles in the proliferation and differentiation of alveolar epithelial cells.

TTF-1, also known as Nkx2.1, is detected as early as E8 in mouse endodermal cells and is identified as the earliest marker of the lung. TTF-1 regulates the expression of all the surfactant protein genes, including SP-A, B, C and D. Mice deficient of TTF-1 have abnormal lungs, which fail to express all the surfactant proteins and have significantly reduced collagen type IV and integrins [6].

HNF-3β is highly expressed in ciliated and columnar bronchial epithelial cells and AEC II during development. HNF-3β induces the expression of various epithelially restricted genes in the lung, including TTF-1 [7], SP-B [8] and CCSP [9,10], in association with the differentiation of lung epithelial cells such as AEC II and Clara cells.

HFH-4 is expressed in the epithelium during fetal lung development, and in basal and ciliated epithelial cells in the adult lung [11]. HFH-4 induces the expression of β-tubulin IV in the pseudoglandular stage, and promotes the differentiation of ciliated epithelial cells.

Other transcription factors, such as GATA-5, GATA-6, and Fox, are also important for the differentiation of epithelial cells in the lung [1]. The expression of these transcription factors decreases with the progression of development and is only restricted in subsets of Clara cells and AEC II at the late stage of development.

### 1.3 Epithelial-mesenchymal interactions

The interactive signaling between epithelial and mesenchymal cells plays an important role in morphogenesis and cell differentiation in the developing lung. Removing the mesenchyme from the embryonic lung rudiment impairs the branching morphogenesis [12]. Lung mesenchyme has the ability to induce branching morphogenesis in non-lung epithelium such as the salivary gland [13] and embryonic trachea, in which mesenchyme has been removed [14,15]. However, non-lung mesenchyme was only able to induce a bud in gut endoderm and these buds had no further branching [14]. Besides its function in determining the epithelial patterning, mesenchyme can also dictate the differentiated phenotype of the epithelium [16].

The communication between mesenchyme and epithelium is mediated by many growth factors. These growth factors are precisely regulated in a temporal and spatial manner during fetal lung development. Fibroblast growth factors (FGFs) and their receptors are among the best characterized growth factors. FGF10 is located in the mesenchyme around distal lung epithelial tips. It binds to the FGFR2b on the epithelial cells and transmits a signal to induce the initiation of the lung bud [17-22]. Recombinant FGF10 alone can induce budding in the lung epithelial explants whose mesenchyme has been removed [18]. Mice deficient of FGF10 or FGFR2b expression have severe abnormalities in lung development [22,23]. The expression of FGF10 and bud formation is regulated by retinoid acid because an antagonist of retinoid acid completely prevents the formation of lung buds from foregut explants [24]. Retinoid acid accelerates the development of the alveolar tree and promotes the expression of surfactant proteins and enzymes for the synthesis of surfactant lipids [25].

On the other hand, pulmonary epithelial cells also influence the proliferation and differentiation of mesenchymal and vascular cells [3]. The epithelial cells secrete vascular endothelial growth factors (VEGF), which binds to its receptors, flk and flt, in the progenitor cells of mesenchyme, and at least in part, regulates pulmonary vasculogenesis [26]. Similarly, Platelet-Derived Growth Factor (PDGF), which is expressed in the epithelial cells, stimulates the differentiation and proliferation of myofibroblasts in the developing lung [27]. Sonic Hedgehog (Shh) is a growth factor expressed in the developing epithelium, most abundantly in terminal buds. Its receptor Patched-1 (Ptc) is located in the mesenchymal cells. The interaction between Shh and Ptc is required for lung bud formation [28-30]. The overexpression of Shh in AEC II with a SP-C promoter disturbs the formation of alveoli by increasing the proliferation of mesenchymal cells, but not epithelial cells [28].

Other growth factors, such as transforming growth factors (TGF-β) and epidermal growth factor (EGF) are also involved in the epithelial-mesenchymal interactions and play essential roles in lung development [31].

## 2. Pleiotrophin

Pleiotrophin (PTN) is an 18 kDa heparin-binding cytokine and shares 50% sequence homology with midkine [32]. PTN has two beta-sheet domains that bind to heparin and extracellular matrix with high affinity [33]. The amino acid sequence of PTN is highly conserved among different organisms.

PTN was first identified as a growth factor in the bovine uterus [33] and as a neurite outgrowth promoting factor in the neonatal rat brain [34]. In comparison with midkine, which is regulated by retinoid acid [35], PTN does not respond to retinoid acid but can be up-regulated by PDGF in primary hepatic stellate cells [36]. The mRNA expression of PTN is significantly up-regulated in some organs in midkine deficient mice, suggesting that PTN and midkine have functional redundancy [37]. In fact, PTN and midkine do share multiple functions. They both regulate the neurite outgrowth, modulate cancer development, enhance cell proliferation and migration, inhibit apoptosis, and have important roles in epithelial-mesenchymal interactions during organogenesis [38,39].

### 2.1 The expression of Pleiotrophin

PTN is expressed in a temporal and cell type-specific manner in order to precisely restrict its functional activities at the right time and at the right site. During mouse embryogenesis, PTN is highly expressed in the central and peripheral nervous systems, in organs undergoing branching morphogenesis including the salivary glands, lung and kidney, digestive and skeletal systems, sense organs and facial processes, and limbs [40]. The expression of PTN is detected as early as embryonic day 9 and peaks in the late stage of embryogenesis (shortly after birth) [41,42]. PTN is mainly located in the basement membrane of the developing epithelium and in mesenchymal tissues undergoing remodeling, suggesting that it may play an important role in mesenchymal-epithelial interactions. In the adult stage, PTN expression is mainly restricted to the central nervous system [41,43].

### 2.2 Functions of PTN

PTN is highly expressed in fetal bone cartilage and implicated in bone formation and remodeling [44]. During the early stages of osteogenic differentiation, PTN is synthesized by osteocytes and located at sites where new bones are formed [44,45]. Exogenous PTN, but not midkine, promotes the chondrogenesis in micromass culture of chicken limb bud mesenchymal cells [46]. As a growth factor that stimulates the proliferation and differentiation of osteoblastic MC3T3-EL cells, PTN promotes the bone morphogenetic protein (BMP)-induced osteogenesis at a high concentration and has an opposite effect at a low concentration [47,48]. Targeted overexpression of PTN in mice promotes bone growth and maturation during the early stages of bone development. However, the effect is diminished with advanced age and the generated bones are more brittle compared to the wild type [48].

Kidney development involves repeated branching morphogenesis and prominent interactions between mesenchyme and epithelium. In the embryonic kidney, PTN is present in the basement membrane surrounding the developing ureteric bud. Recombinant human PTN increases the branching morphogenesis of the cultured uteric bud, in the presence of glial cell-derived neutrophoic factor (GDNF) [49]. In the absence of GDNF, PTN still has the ability to induce the branching morphogenesis of uteric cells [49]. These studies suggest that PTN is one of the key modulators of branching morphogenesis in the kidney.

PTN is up-regulated in the injured rat brain cells [50]. After ischemia exposure, much higher PTN levels have been observed in macrophages, endothelial cells and astrocytes in the mouse brain, especially in the area with high neovasculogenesis activity. This result indicates that PTN participates in neurovascular formation during development. PTN up-regulation is also observed in the dermis after an incisional wound in the rat skin [51]. Additionally, local delivery of PTN in dog fibrin glue after angioplasty injury, significantly increases the rates of re-endothelialization. This effect is mainly due to the stimulation of endothelial cell angiogenesis, and the promotion of smooth muscle cell proliferation [52]. All of these studies suggest that PTN plays a role in injury repair.

PTN levels are also much lower in adult tissues than these in fetal tissues. However, PTN is overexpressed in a number of cancers, such as human breast cancer [53-55], melanocytic tumors [56,57], and glioblastoma [58-61]. As a heparin-binding cytokine, PTN acts as a growth factor to promote cell growth in cells transformed by the v-sis oncogene [33]. The function of PTN in tumor angiogenesis has been addressed to some extent. SW-13 cells transformed by the ectopic expression of PTN exhibit a much higher growth rate and a higher density of microvessels [62]. The nude mice injected with PTN-transformed NIH 3T3 cells have a higher degree of tumor angiogenesis [63]. This effect could be blocked by a dominant negative PTN [64]. PTN also increases the endothelial cell proliferation and tube formation [50]. These studies strongly suggest that PTN is an angogenic factor during tumor formation and a potential target for cancer therapy. PTN also functions as a mitogen for endothelial cells [50,51], epithelial cells and different fibroblast cell lines [33]. The function of PTN can be extended to other aspects, such as regulating the long-term potentiation by controlling the neurite cell outgrowth [65].

### 2.3 PTN regulatory pathways

PTN signals through three cell surface receptors, syndecan-3, anaplastic lymphoma kinase (ALK), and protein tyrosine phosphatase receptor (RPTPβ/ζ).

Syndecan-3 belongs to the syndecan family and is a transmembrane protein. Its extracellular domain contains 3 glycosaminoglycan attachment sites [66]. The binding of PTN with syndecan-3 induces neurite outgrowth of embryonic neurons [67]. Heparitinase, which cleaves the heparin sulfate chain and disrupts the binding of PTN, inhibits PTN-induced neurite outgrowth. Anti-syndecan-3 antibodies have a similar effect. Additionally, the overexpression of syndecan-3 in N18 neuroblastoma cells significantly increases the PTN-induced neurite outgrowth. The PTN/syndecan-3 pathway is possibly mediated by the c-Src, which binds to the intracellular domain of syndecan-3 and subsequently alters the activity of cortactin [68].

ALK is a receptor tyrosine kinase highly expressed in the developing nervous systems and in some tumor cells [69,70]. It shows a similar expression pattern as PTN in different cell lines [71]. Upon the binding with PTN, ALK phosphorylates Ras protein or Akt, and thus activates the Ras-MAPK or the PI_3_K-Akt signaling pathway. This sequentially stimulates cell proliferation and mitogenesis, and inhibits apoptosis [58,71]. However, a recent study has shown that ALK does not directly bind with PTN, but is one of the substrates of RPTPβ/ζ [72].

RPTPβ/ζ is a transmembrane tyrosine phosphatase, which is composed of a cytosoplasmic portion that carries protein tyrosine phosphatase activity, a transmembrane region, and an extracellular domain containing chondroitin sulfate for ligand binding [73]. The extracellular part of RPTPβ/ζ also possesses a carbonic anhydrase-like domain, a fibronectin III-like domain, and a glycine-serine rich domain [73]. These domains interact with the adhesion molecules and mediate the cell-cell adhesions.

PTN is identified as the first natural ligand for the transmembrane tyrosine phosphatase receptor. It binds to the chondroitin sulfate portion of RPTPβ/ζ with high affinity [74]. In U373-MG glioblastoma cells, the binding of PTN with RPTPβ/ζ inactivates the receptor, and thus significantly increases the tyrosine phosphorylation of β-catenin [75,76]. Phosphorylated β-catenin rapidly dissociates from E-cadherin and accumulates in the cytoplasm. The disassociation of β-catenin from E-cadherin disrupts the cell-cell adhesion and possibly promotes cell migration. Another downstream target of the PTN/RPTPβ/ζ is β-adducin [77,78]. Recently, the Src family member, Fyn has been identified as an additional substrate of the PTN/RPTPβ/ζ signaling pathway [79].

RPTPβ/ζ is broadly expressed in almost all of the human breast cancer cells lines, and it plays an important role in the adhesion and migration of tumor cells [80]. Since the PTN pathway through ALK is also mediated through RPTPβ/ζ, the signal through RPTPβ/ζ may be the main regulatory pathway for PTN to regulate cell growth, proliferation, migration, and mesenchymal-epithelial transition [76].

### 2.4 PTN knockout mice

Two research groups have generated PTN knockout mice to investigate the functions of PTN. PTN deficient mice are anatomically normal. However, these mice exhibit enhanced hippocampal long-term potentiation [65]. Deficiency of PTN results in an increased proliferation rate of neuronal stem cells in the adult mouse cerebral cortex [81]. This is consistent with the observation that exogenous PTN reduces the neuronal stem cell proliferation through inhibiting the expression of FGF-2 and promotes cell differentiation [81].

The few abnormalities shown by the PTN knockout mice seem to be inconsistent with the crucial roles of PTN in the proliferation, differentiation and migration of various cells. This may be partially due to the functional redundancy between PTN and midkine. Lack of PTN expression might somehow be compensated by midkine. To address this issue, one group has produced PTN and midkine double knockout mice. These mice show a reduced expression of beta-tectorin and have serious auditory deficits [82]. Additionally, they exhibit significantly reduced reproduction abilities [83].

Transgenic mice overexpressing PTN show abnormalities in brain and bone formation and remodeling. PTN overexpressing mice are morphologically normal, but have attenuated hippocampal long term potential [84]. Specifically overexpressing PTN in osteoclasts under the control of human osteocalcin promoter increases bone mass in female mice, but not in male mice [85,86]. These mice also have advanced bone growth during the early developing stage, damaged fracture healing, and delayed callus formation [48].

### 2.5 PTN and fetal lung development

There are relatively less reports on the PTN functions in the lung. Earlier studies have shown that PTN is expressed in the fetal lungs and some lung cancer cells [40,42]. PTN expression in the lung appears to be independent of midkine expression [37]. During our efforts in gene expression profiling of lung development, we have identified 583 differentially expressed genes, which can be classified into seven clusters [87]. Most of the genes in cluster 5 are related to cell differentiation and development and are highly expressed in the late stages of fetal lung development. PTN is one of the genes in this cluster. PTN is mainly localized in the mesenchymal cells surrounding the developing epithelia and is enriched in fibroblasts [87,88]. Consistent with its role in vasculogenesis and tumor agogenesis [89], PTN expression is also observed in endothelial cells in the developing lung. In contrast, the PTN receptor RPTPβ/ζ, is expressed in the airway epithelial cells at the late stages of fetal lung development. This suggests that PTN may mediate mesenchymal-epithelial interactions.

PTN has multiple functions in fetal lung development. At the early stage of development, PTN is essential for branching morphogenesis [88]. The silencing of PTN in fetal lung organ culture results in the reduction of terminal bud counts, but has no effects on the sizes of terminal or inside buds. At the late stages of fetal lung development, PTN stimulates the proliferation of fetal alveolar epithelial type II cells. However, it arrests the trans-differentiation of fetal alveolar epithelial cell type II cells to type I cells [88]. Furthermore, the addition of PTN also accelerates wound healing of the injured fetal type II cell monolayers [88]. This effect is mediated through PTN secreted by fibroblasts since a similar result is observed in the co-culture of fetal type II cell monolayers with fibroblasts. Anti-PTN antibodies can block the effect caused by fibroblasts.

In fetal type II cells, PTN exerts its effects via cross-talk with Wnt/β-catenin signaling [88]. This is supported by the following evidence: (i) Stimulation of fetal type II cells with PTN increases tyrosine phosphorylation of β-catenin; (ii) PTN causes β-catenin nuclear translocation; and (iii) PTN increases LEF/TCF transcriptional activity as determined by TOPflash reporter assay. Delta-like homolog (Dlk1) is a member of the Notch/Delta/Serrate family and initiates Notch signaling. Dlk1 is negatively regulated by PTN signaling, which requires the co-activation of the Wnt pathway [88]. CHIP analysis reveals that Dlk1 is a direct target of the LEF/TCF transcription factor [88]. These observations suggest that PTN acts via Wnt/β-catenin and Notch pathways.

## 3. Wnt signaling pathway

Wnt is a family of growth factors, which play important roles cell fate determination during lung development. Wnt has at least 19 isoforms, which bind to frizzleds and trigger three intracellular signaling pathways: the canonical Wnt/β-catenin signaling pathway, the non-canonical Wnt/Ca^2+ ^pathway, and the WNT/Planar Cell Polarity (PCP) pathway. The most important pathway of Wnt signaling is the canonical signaling pathway through β-catenin. The binding of Wnt to frizzleds inhibits the activity of glycogen synthase kinase 3β (GSK-3β) and thus stabilizes β-catenin in the cytoplasm. β-catenin accumulates in the cytoplasm and translocates into the nucleus, where it binds to TCF/LEF transcription factors to stimulate the transcription of its downstream genes, such as N-myc, bone morphogenetic protein 4 (Bmp4), and FGF, etc [90].

### 3.1 Wnt and β-catenin expression during fetal lung development

The expression of Wnts and β-catenin are precisely regulated during fetal lung development. *In situ *hybridization reveals that Wnt2 is highly expressed in the fetal lung, and its expression is restricted to mesenchymal cells [91]. In E12.5 to E16.5 mouse lung, Wnt11 expression is observed in epithelial and mesenchymal cells [92], while Wnt7b is only localized in distal and proximal bronchial epithelial cells [93]. Wnt5a expression is barely detectable in a E12 mouse lung, and reaches a high level in E16 in both epithelial and mesenchymal cells. In E18, Wnt5a is mainly localized in airway epithelial cells [94]. Wnt3a expression is expressed in AEC II and some ciliated airway epithelial cells in the adult human lung [95].

β-catenin is expressed in the airway and alveolar epithelial cells during fetal lung development. β-cateinin nuclear expression is especially high in pre-alveolar acini budding from respiratory airways [96]. From E14.5 to E17.5, cytoplasmic and nuclear expression of β-catenin is also found in the primordial and alveolar epithelial cells, and adjacent mesenchymal cells, indicating that the β-catenin signaling may be activated in these cells [96]. The cytoplasmic and nuclear β-catenin level decreases in the mesenchyme after E13.5 [97]. TCF and LEF have a very similar expression pattern as β-catenin during fetal lung development [97]. TCF1 proteins are present in both epithelial and surrounding mesenchymal cells from E10.5 to E17.5. LEF1 protein expression is high in adjacent mesenchyme but low in proximal epithelium. TCF3 and TCF4 proteins are nearly expressed in all kinds of cells, including proximal and distal epithelial cells, and mesenchymal cells from E11.5 to E17.5 [97].

The mesenchymal localization of Wnt ligands and epithelial localization of β-catenin suggest the possible role of Wnt signaling in epithelial-mesenchymal interactions, which are crucial for normal lung morphogenesis, growth, and cell fate determination. Since β-catenin nuclear localization is mainly observed in developing epithelial cells, Wnt canonical signaling may mediate the epithelial proliferation or differentiation.

### 3.2 Wnt signaling in lung morphogenesis

Recently, the transgenic and knockout mice studies have revealed important roles of Wnt signaling in lung morphogenesis. Wnt5a conditional knockout is fatal and results in abnormal distal lung morphogenesis, which is characterized by the hypercellular and thicker intersaccular walls [94]. However, Wnt5a knockout does not affect the vascular distribution and maturation.

Wnt2/2b signaling is essential to specify the lung progenitors in the foregut endoderm [98]. Loss of Wnt2 results in dilated endothelial vasculature, decreased cell proliferation, and down-regulation of the genes crucial for normal lung development. Mouse double deficiency of Wnt2 and Wnt2b exhibits an underdeveloped lung which shows no trachea budding at E9.5, lacks the expression of TTF-1 (a transcription factor crucial for epithelial cell differentiation), and P63 (an esophagus epithelial marker) [98].

The lungs from Wnt7b^lacZ ^mice, which replace the exon 1 with lacZ, exhibit a smaller and collapsed appearance and fail to inflate properly. These mice die shortly after birth [99]. Another defect in the Wnt7b knockout lung is hypoplasia, which is shown by extremely thinner distal mesenchyme. Additionally, smooth muscle α-actin (α-SMA) expression is abnormal in Wnt7b knockout mice. Since smooth muscle cells are differentiated from mesenchymal cells, these studies indicate that Wnt7b affects lung morphogenesis possibly through the regulation of mesenchymal cells.

Deletion of β-catenin in the embryonic mesenchyme leads to shortened trachea, decreased branching, and reduced peripheral mesenchyme [100]. However, the sub-epithelial mesenchyme is not affected. On the other hand, deletion of β-catenin in epithelial cells using SP-C promoter impairs lung morphogenesis, arrests the differentiation of alveolar epithelial cells, and leaves the lung containing mainly conducting airways [101]. Consistently, hyperactivating β-catenin in epithelial cells of the developing lung causes enlarged air space, atypical expression of alveolar type II cells, and goblet cell hyperplasia. And this effect is possibly through the down-regulation of Foxa2 expression in the epithelium [102]. However, further work is required to elucidate the molecular mechanism of this process.

### 3.3 Wnt signaling in cell differentiation and proliferation

The action of Wnt signaling on the lung morphology is mainly achieved by the regulation of proliferation, differentiation, and apoptosis of the lung cells. The regulation of lung cell proliferation by Wnt signaling is well coupled with cell differentiation. The signals that increase the proliferation of progenitor cells normally arrest the differentiation of these cells.

Wnt is important for the cell proliferation and differentiation during fetal lung development, although how Wnt proteins regulate the lung development is still not clear. Wnt7b promoter is regulated by TTF-1 [93], a known transcription factor regulating epithelial cell differentiation in the developing lung. This finding suggests a possible molecular mechanism of TTF-1 in regulating the lung epithelial differentiation.

Wnt7b^lacZ ^mice do not show abnormal differentiation of some epithelial cells including Clara cells, and alveolar type II cells. However, alveolar type I cell differentiation is delayed in Wnt7b^lacZ ^mice, suggesting that Wnt7b may be important for late epithelial cell differentiation. Wnt7b knockout significantly reduces the proliferation of mesenchymal cells on E12.5 but not on E14.5. However, the proliferation of epithelial cells is not affected [99]. The results indicate that Wnt7b is a regulator for mesenchymal cell proliferation in the early developing lung. In addition, apoptosis increases significantly in the vascular smooth muscle and epithelium following Wnt7b deprivation. However, another Wnt7b knockout mouse, Wnt7b^D3^, in which exon 3 is deleted, shows decreased proliferation of both epithelial and mesenchymal cells without perturbing cell differentiation and lung patterning [103]. Interestingly, the development of smooth muscle in these mice is normal. These results are controversial with other findings that Wnt7b/β-catenin signaling is necessary for the smooth muscle cell development [104,105].

Hyperactivation of β-catenin specifically in lung endoderm leads to the increased amplification of distal lung progenitor cells and the shortage of fully differentiated lung cell types [106]. Activation of β-catenin signaling only in epithelial cells causes ectopic differentiation of AEC II [102]. Additionally, conditional knockout β-catenin in mesenchyme increases the proliferation and Fgf10 expression in parabronchial smooth muscle cells (PSMC). However, the differentiation of this group of cells is not affected [100]. All these results indicate that β-catenin signaling is essential for normal epithelial differentiation.

Wnt5a normally activates a non-canonical pathway and inhibits the canonical β-catenin signaling [107]. Conditional knockout of Wnt5a caused a significant increase in lung cell proliferation without interfering with cell differentiation [94]. However, Wnt5a could also induce a canonical β-catenin signaling in Usual Interstitial Pneumonia (UIP) lung fibroblast and promotes the fibroblast proliferation [108].

### 3.4 Wnt signaling and lung diseases

In addition to its role in lung development and morphogenesis, Wnt signaling pathways are also linked to the pathogenesis of several lung diseases. The dysregulation of Wnt signaling in adult lung causes lung cancer, fibrosis, and inflammation [109]. Hyperactivation of β-catenin, caused by mutations of β-catenin, APC, and axin in lung epithelium induces lung tumors [102]. β-catenin is overexpressed and activated in many lung cancer cells. Wnt/β-catenin could become targets for a novel therapeutic strategy for lung cancers.

Fibrosis is a crucial process during tissue repair after an injury. Wnt signaling is activated in the lungs of the patients with idiopathic pulmonary fibrosis [95] and animals with bleomycin-induced pulmonary fibrosis [110]. Hyperactivation of Wnt signaling pathway is suggested as one of the main reasons which causes abnormal fibroblast proliferation and excess extracellular matrix deposition during pulmonary fibrosis. Additionally, Wnt signaling also induces the overexpression of fibrosis regulators such as metalloproteinase and matrilysin [109].

Bronchopulmonary dysplasia (BPD) is a chronic lung disease in infants. BPD is characterized by lung injury resulting from mechanical ventilation and oxygen exposure, or from defects in lung development. Wnt signaling is activated during hyperoxia-induced neonatal rat lung injury, suggesting its role in BPD [111].

## 4. Summary

Defects in pulmonary development normally lead to numerous lung diseases. PTN is a growth factor differentially expressed during fetal lung development. Wnt/β-catenin pathway is involved in epithelial-mesenchymal interactions during lung development. PTN and Wnt signaling pathways are partially overlapped and linked to Notch pathway via Dlk1. Although several signaling pathways have been identified to regulate normal lung development, less is known about the cross-talking among these signaling pathways. Several downstream genes of the Wnt signaling have been identified including Dlk1, TTF-1, BMP4, c-myc, and Axin II. How these genes are properly turned on/off to regulate lung development is not fully understood. The elucidation of roles of PTN and Wnt signaling in fetal lung development and its regulatory pathway may offer opportunities in the development of new therapeutic strategies and drugs to resolve the disorders associated with fetal lung development.

**Figure 1:**
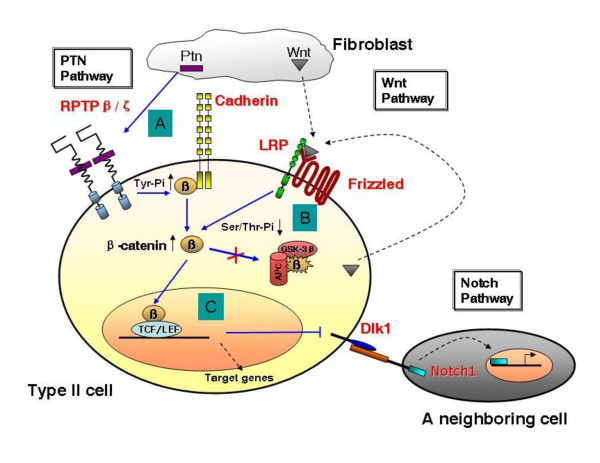
**PTN, Wnt and Dlk1 control alveolar cell proliferation and differentiation in synchrony**.

Finally, we propose the following model for PTN signaling and its cross-talk with Wnt signaling (Fig. 1). (*A*) PTN is secreted by fibroblasts and binds to the receptor protein tyrosine phosphatase β/ζ (RPTP β/ζ). This action inactivates RPTP β/ζ, which results in an increase of the phosphorylation of β-catenin on its tyrosine residues (Tyr-Pi) and the release of β-catenin from cadherins. (*B*) In the absence of Wnt ligands, β-catenin is marked for destruction by proteasomal degradation via its serine/threonine phosphorylation (Ser/Thr-Pi) by glycogen synthase kinase 3β (GSK-3β). The activation of Wnt signaling leads to a decrease in Ser/Thr-Pi, preventing the degradation of β-catenin. (*C*) The binding of nuclear β-catenin with T cell factor/lymphoid enhancer factor (TCF/LEF) transcription factors depresses *Dlk1*, resulting in the inactivation of Notch signaling in a neighboring cell (either an undifferentiated columnar cell or a type I cell). The future directions (dashed lines) include: which Wnt(s) secreted by fibroblasts and/or type II cells activates the Wnt pathway? What are other target genes of TCF/LEF (either depressed or activated)? What signaling does Dlk1 initiate? Further investigations will answer these questions in the near future.

## Competing interests

The authors declare that they have no competing interests.

## Authors' contributions

TW drafted the manuscript. LL helped to draft as well as revised the manuscript. All authors read and approved the final manuscript.
